# Neutrophils: Linking Inflammation to Thrombosis and Unlocking New Treatment Horizons

**DOI:** 10.3390/ijms26051965

**Published:** 2025-02-25

**Authors:** Haokun Li, Wenbo Shan, Xi Zhao, Wei Sun

**Affiliations:** Department of Molecular Biology, College of Basic Medical Sciences, Jilin University, Changchun 130021, China; lihk22@mails.jlu.edu.cn (H.L.); shanwb24@mails.jlu.edu.cn (W.S.); zhaoxi23@mails.jlu.edu.cn (X.Z.)

**Keywords:** neutrophil, inflammation, thrombosis, stroke

## Abstract

Neutrophils play a key role in inflammatory responses and thrombosis, but their complex interactions in disease pathogenesis are not fully understood. This review examines the multifaceted roles of neutrophils, focusing on their activation, cytokine release, and formation of neutrophil extracellular traps (NETs), which contribute to host defense and thrombosis. We discuss the interaction between inflammation and coagulation, the direct effect of neutrophils on thrombus stability, and their involvement in pathological thrombotic diseases. The therapeutic potential of neutrophil drug loading in the treatment of thrombosis, as well as the clinical implications and future research directions, are highlighted. The aim of this review is to gain insight into the critical neutrophil–inflammation–thrombus axis and its potential as a therapeutic target for thrombotic diseases and to suggest possible directions for neutrophil-loaded drug therapy for thrombosis.

## 1. Introduction

Neutrophils serve as a crucial link connecting inflammation and thrombosis, a topic that has been extensively studied in recent years. The inflammatory response is a complex physiological mechanism that is activated when the body encounters exogenous or endogenous stimuli. This response aims to restore homeostasis through the actions of phagocytes and cytokines, which initiate the innate immune response.

When cytokines are produced in excessive amounts, a series of events occur. Vascular permeability increases, and the inflammatory environment can push the blood into a hypercoagulable state. This hypercoagulability predisposes the body to thrombosis. Thrombosis, defined as the formation of blood clots or solid aggregates within the blood, can be triggered by various factors, such as the interaction between platelets and cytokines. Platelets play a multifaceted role in the body. They are involved in hemostasis and host defense and are capable of producing a wide range of inflammatory mediators, including growth factors, cytokines, and chemokines [1].

Neutrophils play a key role in host defense and thrombosis. Neutrophil extracellular traps (NETs) are a key component in this complex interplay. NETs are fibrous network structures that contain DNA, histones, myeloperoxidase, neutrophil elastase, and cathepsin G, which are released from neutrophils [2]. These structures not only capture pathogens and kill bacteria but also play a significant role in promoting the prothrombotic state [3].

Specifically, NETs promote the prothrombotic state and further activate platelets. Activated platelets can then bind to neutrophils. This binding occurs when P-selectin on the platelets attaches to the neutrophil surface receptor PSGL-1, stimulating neutrophils to release more NETs [4]. The interaction between thrombi and inflammation can be regulated by the complex interaction between NETs and cytokines released by neutrophils and platelets, as extensively discussed in recent research.

There are different types of thrombosis. Arterial thrombosis is rich in platelets and often forms adjacent to or around ruptured atherosclerotic plaques, typically occurring in high-shear marginal flow conditions. In contrast, venous thrombosis is rich in fibrin and red blood cells and usually occurs in slow shear flow.

**Figure 1:**
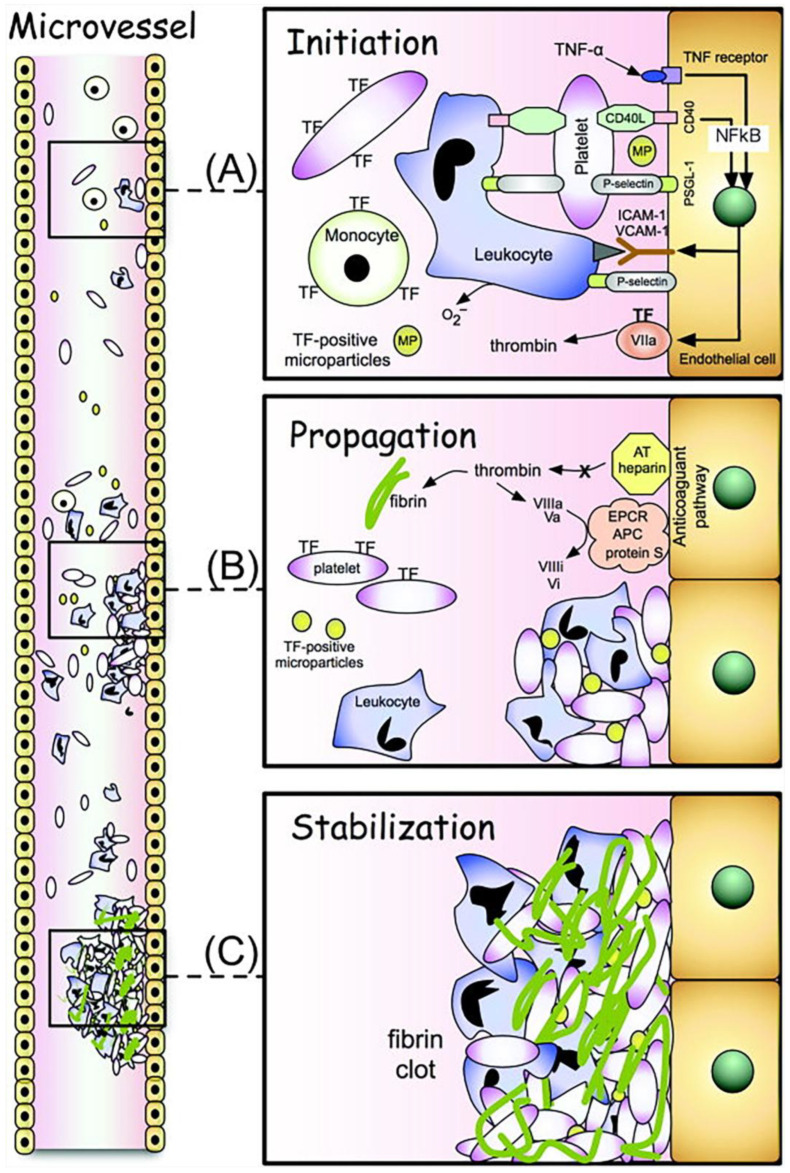
Stages of thrombus formation caused by inflammation. (**A**): Initiation phase: In the absence of collagen exposure, a thrombus is likely initiated by the recruitment of tissue factor (TF)-expressing blood cells (platelets, leukocytes) and TF-positive microparticles, as well as an increased expression of TF by endothelial cells. Cytokines (e.g., TNF-α) and CD40/CD40L interactions can elicit the expression of the TF and adhesion molecules (ICAM-1, VCAM-1, P-selectin) via NFkB-mediated, transcription-dependent mechanisms. Platelet-endothelial cell and platelet-leukocyte adhesion are mediated by P-selectin (platelet)-PSGL-1 (endothelial cell or leukocyte) interactions. ICAM-1, VCAM-1, and P-selectin expression by activated endothelium mediates the adhesion of leukocytes in the vasculature. (**B**): Propagation phase: Thrombin generation from the extrinsic (TF-VIIa) pathway, activation of the intrinsic pathway, and the recruitment of additional TF-bearing platelets, TF-positive microparticles, and leukocytes greatly amplifies thrombus formation. Inhibition of the activated protein C pathway and antithrombin (AT)-heparin system further increases thrombin generation. (**C**): Stabilization phase: The fibrin deposition resulting from the action of thrombin on fibrinogen serves to stabilize the thrombus. Reproduced with permission from Inflammatory Bowel Diseases, Copyright 2009, Yoshida, H. [9].

Two main mechanisms mediate vascular homeostasis and thrombosis, depending on vascular injury or vascular architecture [5]. One mechanism is mediated by collagen, and the other is dependent on tissue factor (TF). TF is the major initiator of the exogenous coagulation cascade. When stimulated with platelet-activating factor (PAF) or granulocyte–macrophage colony-stimulating factor, TF is translocated to the cell surface, triggering thrombin production and ultimately leading to thrombosis (Figure 1) [6,7].

**Figure 2:**
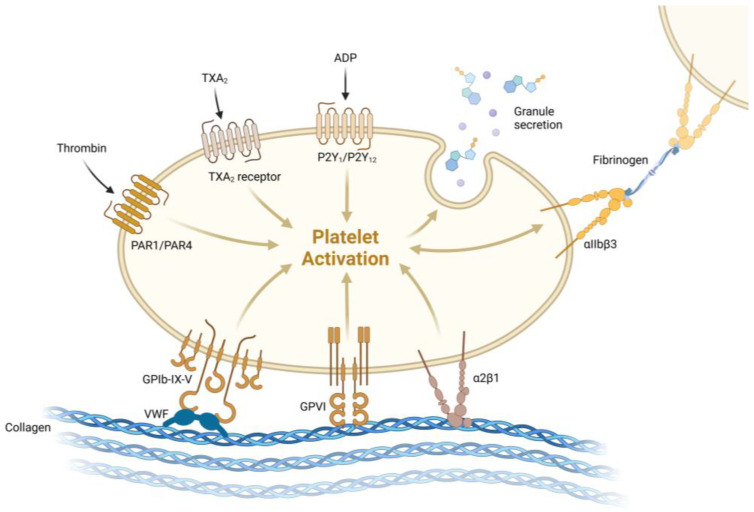
Platelet activation. GPVI is expressed by platelets and has high affinity for collagen. Figure created using Biorender.

During normal hemostasis, the endothelial cell layer may be damaged, exposing collagen in the subendothelial space. Platelets interact with collagen and von Willebrand factor (vWF) through their glycoproteins GPVI and GPIb/V/IX. Activated platelets secrete aggregation mediators such as thromboxane A2, ADP [8], and large amounts of vWF polymers, along with serotonin and thrombin, to recruit other circulating platelets (Figure 2). Deeper tissue damage results in the release of TF from smooth muscle advent, itia, and pericytes. TF then mediates the conversion of prethrombin to thrombin, the production of fibrin, and the activation of the coagulation cascade. In addition to the above mechanisms, there are other factors contributing to thrombosis. For example, one mechanism involves the interaction of platelet abnormalities with cytokines, leading to the formation of thrombi.

Given the intricate and multifaceted roles that neutrophils play in both thrombosis and inflammation, as well as their complex interactions with platelets, it is imperative to delve deeper into these relationships. Understanding how neutrophils contribute to the inflammatory cascade and thrombotic processes, and elucidating the mechanisms through which they interact with platelets, could pave the way for novel therapeutic strategies. These insights may ultimately lead to more effective and targeted treatments for thrombotic disorders, potentially improving patient outcomes and reducing the burden of thrombosis-related morbidity and mortality.

## 2. Neutrophils in the Inflammatory Response

As the most abundant circulating leukocytes (40–70% of human leukocytes) [10], neutrophils serve as indispensable sentinels of innate immunity and primary effectors in acute inflammation. Their recruitment to sites of injury or infection follows a tightly regulated three-step cascade: (1) selectin-mediated rolling along the vascular endothelium, (2) integrin-dependent firm adhesion (e.g., CD11b/CD18 binding to ICAM-1), and (3) chemokine-guided transendothelial migration [11,12]. This process is triggered by inflammatory mediators such as IL-8, TNF-α, C5a, and LTB4, which activate endothelial cells to express adhesion molecules and establish chemotactic gradients [11,13]. Upon reaching inflamed tissues, neutrophils deploy three key antimicrobial strategies:

1. Opsonin-dependent phagocytosis (mediated by IgG/C3b receptors);
2. Degranulation releasing cytotoxic enzymes (myeloperoxidase, defensins);
3. NETosis—a programmed cell death mechanism producing chromatin-based extracellular traps laden with histones and proteases [2].

Recent advances reveal remarkable neutrophil plasticity, with distinct subsets (N1 pro-inflammatory vs. N2 anti-inflammatory) differentially regulating inflammation resolution [14]. Intriguingly, neutrophils also bridge innate and adaptive immunity via cytokine secretion (IL-12, IFN-γ) and MHC class II-mediated antigen presentation [15].

Neutrophil lifespan is tightly controlled by a balance between proapoptotic (e.g., Fas/FasL) and antiapoptotic (e.g., GM-CSF) signals, typically limiting survival to <24 h to ensure inflammation resolution [16]. Delayed apoptosis disrupts macrophage-mediated clearance, leading to secondary necrosis and sustained release of DAMPs (e.g., HMGB1), which amplify cytokine storms and exacerbate tissue damage [17]. In thrombotic inflammation, this dysregulated neutrophil accumulation creates a feed-forward loop—persistent inflammation promotes neutrophil recruitment, while neutrophil-derived mediators (proteases, NETs) directly fuel thrombogenesis through fibrin deposition and platelet activation [18].

Notably, neutrophils exhibit dual roles as both protective defenders and pathogenic drivers. While essential for host defense, excessive activation contributes to tissue destruction in chronic inflammatory diseases (e.g., rheumatoid arthritis, ARDS) [19]. Emerging therapeutic strategies targeting recruitment pathways (α4β1 integrin blockade) or NET components (DNase I) show efficacy in preclinical models, highlighting opportunities to modulate neutrophil activity without compromising antimicrobial functions [20].

## 3. Formation and Function of Neutrophil Extracellular Traps (NETs)

Neutrophils, as the first line of defense in the innate immune system, play a critical role in combating microbial infections. One of their remarkable mechanisms is the formation NETs, web-like structures composed of decondensed chromatin decorated with antimicrobial proteins, including histones, myeloperoxidase (MPO), and neutrophil elastase (NE). NETs are released through a unique form of cell death known as NETosis, which can be triggered by various stimuli such as pathogens, inflammatory cytokines, and activated platelets [2]. During NETosis, the nuclear envelope disintegrates, allowing the mixing of nuclear DNA with cytoplasmic granule proteins, followed by the extrusion of this complex into the extracellular space [21]. This process not only traps and neutralizes pathogens but also contributes to the resolution of inflammation by degrading virulence factors and preventing microbial dissemination [22].

Beyond their antimicrobial role, NETs have been implicated in a range of pathological conditions, including autoimmune diseases, thrombosis, and cancer metastasis. For instance, excessive or dysregulated NET formation can damage host tissues, perpetuate inflammation, and promote the development of autoimmune diseases such as systemic lupus erythematosus (SLE) [23]. Additionally, NETs have been shown to play a pro-thrombotic role by providing a scaffold for platelet aggregation and coagulation factor activation [24]. These dual roles of NETs in host defense and thrombosis highlight their complex and context-dependent functions in health and disease.

### 3.1. The Role of NETs in Host Defense

NETs are a crucial component of the innate immune system, playing a pivotal role in host defense against microbial infections. NETs are composed of decondensed chromatin fibers adorned with antimicrobial proteins, such as histones, neutrophil elastase, and myeloperoxidase, which collectively function to trap and neutralize invading pathogens [2]. By forming a physical barrier, NETs prevent the dissemination of bacteria, fungi, and viruses, thereby limiting the spread of infection. For example, NETs have been shown to effectively capture and kill Staphylococcus aureus and Candida albicans, highlighting their broad-spectrum antimicrobial activity [25,26], and can even capture HIV and eliminate HIV through peroxidase and alpha defensin [27].

In addition to their trapping function, NETs exert direct microbicidal effects. The high local concentration of antimicrobial proteins embedded within NETs disrupts microbial membranes and degrades virulence factors, enhancing pathogen clearance [22]. Furthermore, NETs can modulate the immune response by acting as a scaffold for the binding of complement proteins and immunoglobulins, thereby amplifying opsonization and phagocytosis [28]. This dual mechanism of physical entrapment and biochemical attack underscores the versatility of NETs in combating infections.

However, the role of NETs in host defense is not limited to extracellular pathogens. Recent studies have demonstrated that NETs can also target intracellular pathogens, such as Mycobacterium tuberculosis, by facilitating their exposure to the immune system [29]. Moreover, NETs contribute to the containment of viral infections by trapping viral particles and preventing their spread [27]. Despite their protective role, excessive or dysregulated NET formation can lead to collateral tissue damage and contribute to chronic inflammatory diseases, highlighting the need for a tightly regulated balance in NET activity [30].

### 3.2. The Role of NETs in Thrombosis Promotion

**Figure 3:**
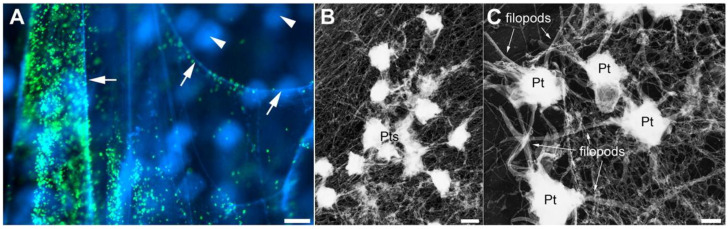
NETs provide a scaffold for platelet adhesion and aggregation. (**A**) Platelets (green) bound to NETs (blue, arrows). Neutrophils (blue, arrowheads)were out of focus and did not bind platelets. (Scale bar, 20 μm.) (**B**) Electron micrograph of platelets (Pts) attached to a fibrous meshwork of NETs. (Scale bar,1 μm.) (**C**) Numerous filopods indicated that platelets (Pt) on NETs were activated. (Scale bar, 0.5 μm.) Reproduced with permission from Proceedings of the National Academy of Sciences, Copyright 2010, Fuchs, T.A. [24].

In addition to pathogenic microorganisms, many factors can stimulate NET production by neutrophils, including LPS, IL-8, PMA [11], IFN, activated platelets, endothelial cells, and plasma from patients with sepsis. NETs have a significant effect on coagulation. Histones specifically activate platelets [31], inhibit activated protein C-mediated coagulation [32], and promote thrombin activation [33]. DNA can activate factor XII and initiate coagulation [34], whereas elastase can breakdown coagulation inhibitors [35]. NETs promote thrombosis mainly by interacting with endothelial cells, platelets, red blood cells, and coagulation factors. Fuchs et al. demonstrated that NETs provide a stimulating environment and scaffold for thrombus formation, during which platelet adhesion, aggregation, and activation occur. NETs can also recruit red blood cells, promote fibrin deposition, and induce red thrombus formation (Figure 3) [24]. Brill et al. confirmed in mice that NETs are the third scaffold structure for deep-vein thrombosis besides fibrin and vWF. Both DNA and histone are involved in the formation of thrombosis, and NETs also provide a new target for the treatment of deep vein thrombosis [36]. Treatment of NETs with DNase can prevent thrombosis by degrading their network structure [37].

NETs can also mediate thrombus formation in Acute Respiratory Distress Syndrome (ARDS). First, the network structure of NETs provides a scaffold for the deposition of platelets, red blood cells, fibrinogen, microparticles, vWF, and other substances, which is conducive to thrombosis [38]. High levels of neutrophil and NET infiltration, platelet aggregation, and microthrombosis have been observed in the pulmonary microvessels of influenza A virus-infected mice [39]. In addition, many components of NETs themselves can participate in blood coagulation. Studies [40] have shown that histone proteins in NETs can stimulate endothelial cells to synthesize and release vWF and activate platelets through TLR-4 to promote platelet aggregation. In septic-induced (ARDS), histones within NETs bind to Thrombomodulin (TM) to inhibit TM-dependent Protein C (PC) activation [41].

Proteolytic enzymes in NETs also suppress the activities of Antigen-Presenting Cells (APCs) and Tissue Factor Pathway Inhibitors (TFPIs) [42]. Studies have reported that Coagulation Factor XII (FXII) can be directly activated by peripherally attached platelets and free DNA (negative charge) in NETs, where it can activate the endogenous coagulation pathway [43]. An autopsy of samples from COVID-19 patients and patients with other lung diseases revealed increased expression of FXII in the lung parenchyma, pulmonary blood vessels, and alveolar space, with FXIIa and NETs colocalizing in the lung tissue of COVID-19 patients, indicating that NETs may induce the activation of FXII [44]. Furthermore, various Damage-Associated Molecular Patterns (DAMPs), such as free DNA and histones, induce the expression of numerous TFs by activating the innate immune system [45]. In sepsis, excessive NETosis exacerbates organ failure by amplifying platelet activation, fibrin deposition, and systemic inflammation; significantly increased levels of TF-rich NETs have been found in both animal models and patients with sepsis-induced ARDS, and blocking NETs can reduce disease progression [46].

In severe COVID-19, NETs contribute to immunothrombosis by serving as scaffolds for thrombus formation, activating coagulation via TF release, and inhibiting thrombomodulin-dependent anticoagulant pathways through histone-mediated mechanisms [47,48]. Elevated NET markers, such as citrullinated histones and MPO, are associated with microvascular endothelial injury and hypercoagulability, especially in adults with respiratory failure. In pediatric populations, MIS-C—a post-infectious hyperinflammatory syndrome—displays distinct thrombotic pathways, where unresolved NETs may act as DAMPs, perpetuating cytokine storms and endothelial dysfunction, even though the virus itself has largely disappeared. Multi-omics studies have shown that the thrombosis mechanism of COVID-19 and MIS-C in children is different from that in adults: in adults, it is mostly driven by fibrin-mediated red blood cell aggregation, while in children, it is more likely to be associated with excessive cytokine release, suggesting that NETs may lead to thrombotic inflammation through different pathways in different age groups [49].

## 4. Neutrophil-Platelet Interactions in Thrombosis

Neutrophils, classically recognized as first responders to infection, are now established as pivotal players in thrombotic processes through dynamic crosstalk with platelets, endothelial cells, and the coagulation cascade. Their dual role in innate immunity and thrombosis is mediated by mechanisms such as neutrophil extracellular trap (NET) formation, expression of procoagulant factors, and bidirectional interactions with platelets. These processes are amplified in pathological contexts including sepsis, cardiovascular diseases, and cancer-associated thrombosis [3,30,34].

### 4.1. Mechanisms of Neutrophil-Driven Coagulation

Neutrophils directly activate coagulation pathways via:

1. Tissue Factor (TF) Expression: Activated neutrophils surface-expose TF, initiating thrombin generation and fibrin deposition. A seminal study by Darbousset et al. demonstrated that neutrophils arriving at sites of laser-induced vascular injury precede platelets, releasing TF and thrombin to nucleate thrombus formation [50].
2. NETosis: NETs—chromatin fibers decorated with histones, NE and MPO—serve as scaffolds for platelet adhesion and amplify thromboinflammation. H3/H4 histones bind von Willebrand factor vWF and platelet receptors, triggering aggregation, while NE degrades anticoagulants like tissue factor pathway inhibitor (TFPI) [51,52].
3. Biomechanical Signaling: Mechanical stress (e.g., arterial stiffness, hypertension) primes neutrophils for NETosis via calpain-PI3K/FAK pathways, linking hemodynamic forces to thrombosis [53]. Flow shear stress increases the intracellular calcium level by activating Piezo1 ion channel, promotes NETosis, and enhances platelet adhesion to NETs [54].
4. Migrasomes: Recently identified neutrophil-derived vesicles adsorb coagulation factors (e.g., prothrombin, FX) and localize to injury sites, acting as platforms for fibrin generation [55].

### 4.2. NETs: A Central Hub in Thromboinflammation

**Figure 4:**
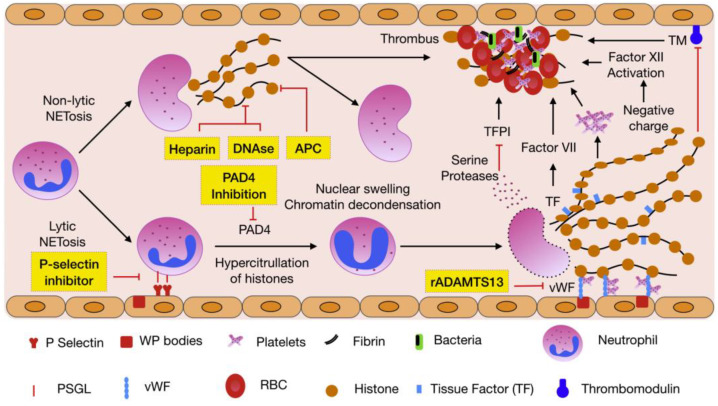
NET formation and its role in thrombosis. Reproduced with permission from Thrombosis Research, Copyright 2018, Kapoor, S. [3].

NETs play an important role in arterial thrombosis (Figure 4). NETs orchestrate thrombosis through multiple convergent pathways:

1. Platelet Activation: NET components (e.g., HMGB1) engage platelet Toll-like receptors (TLR2/4), inducing activation and reciprocal NETosis. This creates a feedforward loop where platelets release polyphosphates and DAMPs to further stimulate neutrophils [56,57].
2. Coagulation Cascade:
  Extrinsic Pathway: NET-associated TF bypasses TFPI inhibition to activate FVII.
  Intrinsic Pathway: Histones and DNA directly activate FXII, fostering thrombin burst [58].
3. Thrombus Stabilization: In myocardial infarction, NETs constitute up to 40% of thrombus volume, correlating with impaired ST-segment resolution and larger infarct size [59].

Dysregulated NETosis underlies immunothrombosis in sepsis-induced multiorgan failure and COVID-19-associated pulmonary thromboinflammation. Therapeutic strategies targeting NET components (e.g., DNase I, PAD4 inhibitors) or NET-platelet interfaces (e.g., TLR4/PSGL-1 blockers) show promise in preclinical models [44,60].

### 4.3. Neutrophil-Platelet Crosstalk: Molecular Mechanisms

Studies have shown that neutrophils through the interaction of platelet and by releasing factor to promote the activation of the coagulation system, so as to accelerate the formation of blood clots [61]. In addition, neutrophils may participate in the process of vascular wall inflammation and vascular remodeling by interacting with vascular endothelial cells [62].Platelets amplify neutrophil recruitment and effector functions through:

1. Receptor-Ligand Interactions:
  P-selectin/PSGL-1 binding induces MAC-1 activation on neutrophils, reinforcing adhesion to GPIbα on platelets [63].
  CD40L-CD40 signaling promotes ROS production and sustains platelet-neutrophil aggregates (PNAs) [64].
2. Soluble Mediators:
  Platelet-derived IL-8, C5a, and CXCL4 enhance neutrophil chemotaxis and NETosis [65].
  Microparticles from activated platelets deliver TF and polyphosphates, amplifying thrombin generation [66].
3. Innate Immune Synergy:
  Platelet TLR2/4 activation by pathogens (e.g., LPS) triggers PNA formation and NET release, critical in sepsis-associated disseminated intravascular coagulation [67].
  TREM-1/CLEC-2 interactions modulate neutrophil effector functions, influencing thrombosis resolution [68,69].

PNAs drive microvascular occlusion in acute lung injury, ischemia-reperfusion injury, and atherosclerosis. Targeting PNA formation (e.g., P-selectin inhibitors) reduces thrombotic burden in animal models [70,71].

In summary, neutrophils orchestrate coagulation through NETs, biomechanical signaling, and migrasomes, bridging innate immunity and thrombosis. Understanding these pathways offers opportunities to mitigate thrombotic complications in inflammatory and vascular diseases.

## 5. Crosstalk Between Inflammation and Coagulation

The interplay between inflammation and coagulation is a tightly regulated process critical for the body’s response to injury and infection. This interaction is not unidirectional; rather, it is a complex and dynamic process where the activation of one system significantly influences the other.

There is a network relationship between inflammation and thrombosis. On the one hand, inflammation promotes blood entry into a hypercoagulable state; on the other hand, the products of thrombosis also cause inflammation. The role of inflammatory cells in thrombosis may be caused by a variety of cytokines and inflammatory mediators secreted by leukocytes and neutrophils and by inflammatory mediators that act on some aspects inside and outside the coagulation system, causing thrombosis [72]. There are intricate links between coagulation and innate immunity, such that neutrophil elastase and thrombin G as well as extracellular ribosomes enhance local proteolytic activity, thereby promoting tissue factor-mediated and FXII-dependent coagulation pathways and modulating the action of the coagulation inhibitor TFPI [73]. This interaction is a driving force in both in vivo coagulation and the growth of intravascular thrombi.

### 5.1. The Inflammatory Response Causes the Coagulation

Inflammation can cause the coagulation reaction. For example, interleukin-1 (IL-1) and tumor necrosis factor-α (TNF-α) secreted by leukocytes and neutrophils can promote fibrinogen aggregation and inhibit fibrinolysis. The anticoagulant state is converted to a precoagulated state. These cytokines stimulate the expression of TF to play a role, and TF is the major initiating factor of the extrinsic coagulation pathway, especially in endothelial cells and monocytes. For example, thrombin can directly activate IL-1α, thus directly linking coagulation and the immune system [74]. The complement system, which is part of the innate immune response, can be activated during inflammation and can contribute to coagulation activation through the generation of C3a and C5a, which can stimulate TF expression. In addition, the role of TNF-α in cerebral ischemia–reperfusion injury has been studied, and it may act by affecting the blood–brain barrier, the inflammatory response, thrombosis, and vascular changes associated with brain injury.

The role of platelets in maintaining vascular integrity at sites of inflammation is currently referred to as the inflammatory hemostatic process [75]. The hemostatic and immunomodulatory functions of platelets are tightly regulated by environmental signals, particularly their interactions with endothelial and innate immune system components. The concept of thrombo-inflammation was originally used to describe the role of platelets in reducing the inflammatory response to cerebral ischemia–reperfusion injury [76]. At present, thrombo-inflammation is more widely used to describe various diseases regulated by crosstalk between thrombosis and inflammation, such as deep vein thrombosis, stroke, and atherosclerosis, and infectious diseases such as sepsis. A common feature of inflammatory thrombotic diseases is the interaction of endothelial cells with the immune and hemostatic systems. In these diseases, inflammation triggers thrombosis, which in turn stimulates an inflammatory response. Interactions between platelets and leukocytes enhance the role of inflammatory endothelial cells, thrombi, and blood in experimental models of inflammation, acute coronary syndrome, ischemic stroke, deep vein thrombosis, and sepsis.

### 5.2. The Coagulation Reaction Can Cause the Inflammatory Response

Similarly, the coagulation reaction can cause the inflammatory response of the body. The coagulation system can modulate the inflammatory response through various mechanisms, including the activation of coagulation proteases such as thrombin, which can interact with specific cell receptors on mononuclear cells or endothelial cells, affecting cytokine production or inflammatory cell apoptosis [77]. Thrombin, for instance, plays a vital role in coagulation’s stimulation of inflammation by converting fibrinogen to fibrin and activating other coagulation factors, while also eliciting responses in various cell types like leukocytes, platelets, and endothelial cells [78]. This crosstalk between the coagulation and inflammation systems is crucial, as it not only helps contain inflammatory activity to the site of injury or infection but also contributes to disease progression, particularly in conditions like sepsis, where the procoagulant stimulus exceeds the capacity of the anticoagulants to control the process. The intricate relationship between inflammation and coagulation is further complicated by the fact that inflammatory mediators can increase platelet count and reactivity, which in turn can propagate the coagulation process [79].

Furthermore, some coagulation factors, such as factor VII, factor Xa, and thrombin, can also have direct proinflammatory effects, such as inducing the expression of TF and cytokines [77]. Many of the coagulation reactions occur on phospholipid surfaces expressing negatively charged phospholipids, where phosphatidylserine seems to play a key role [80]. Inflammatory reactions, such as complement activation or necrosis, could enhance the clotting response by providing the key membrane surfaces on which the initiation and amplification aspects of coagulation could proceed. The natural anticoagulant systems, including the protein C anticoagulant pathway, the tissue factor pathway inhibitor, and the heparin-antithrombin pathway, are necessary to prevent thrombosis [77]. The loss of natural anticoagulant mechanisms can propagate the inflammatory response and cellular apoptosis, further contributing to thrombosis.

### 5.3. Neutrophils and Platelets Represent the Interplay Between Inflammation and Coagulation

The crosstalk between inflammation and coagulation can also be understood in terms of the interaction of neutrophils and platelets. Platelets play a pivotal role in the increase in inflammation and thrombosis observed in sepsis, with their contribution being partially mediated by neutrophils. Upon activation, platelets can direct leukocytes to the infection site, where they form neutrophil–platelet complexes. This interaction not only primes neutrophils for NET release but also bolsters their capacity to eliminate pathogens [81,82]. Conversely, NETs promote further platelet adhesion, activation, and aggregation, thus amplifying the thrombotic response [31].

In mouse models of experimental sepsis, the presence of NETs has been identified as a critical node for platelet aggregation, thrombin generation, and fibrin clot formation, processes that are critical for the progression of sepsis-induced intravascular coagulation. NET-triggered coagulation depends on the interaction of histone H4 in NETs with platelets and the coordinated release of inorganic phosphate [83].

Furthermore, the extent of NET formation in sepsis patients is a significant predictor of the development of disseminated intravascular coagulation (DIC) and associated mortality rates [84]. This correlation underscores the influential role of NETs in the coagulopathy associated with sepsis, highlighting the potential for targeted therapies aimed at modulating NET activity in the treatment of sepsis-induced coagulopathies.

**Figure 5:**
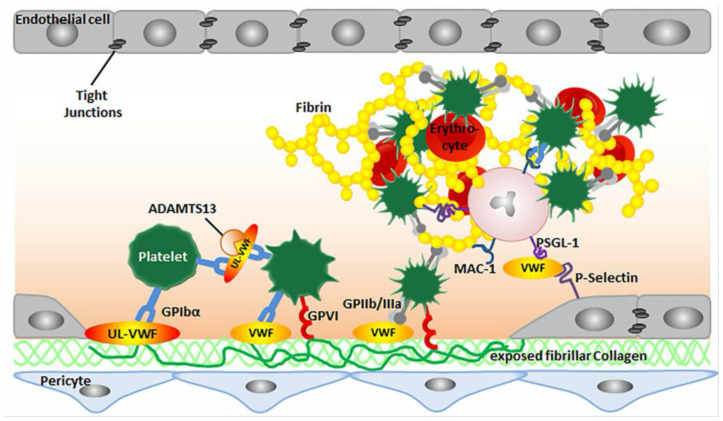
Platelet adhesion and thrombus formation. Reproduced with permission from Stroke. Copyright 2022, De Meyer, S.F. [85].

Vascular endothelial cells are also involved in the interaction between inflammation and coagulation. The vascular endothelium has both procoagulant and anticoagulant properties. During inflammation, the balance shifts toward a procoagulant state due to the release of vWF and TF and the reduction in thrombomodulin and activated protein C activity. Weibel-pad bodies (WP) are specialized endothelial cell-secreted organelles that store factors that regulate vascular hemostasis and local inflammation. Endothelial-cell activation triggers rapid secretion of WPB, leading to the presentation of adhesion molecules involved in leukocyte rolling and platelet trapping (P-selectin and vWF, respectively) on the cell surface (Figure 5).

Recent studies have confirmed that inflammatory mechanisms play an important role in the occurrence and progression of ischemic diseases. The inflammatory response involves the rapid polarization of microglia, the production of proinflammatory cytokines, and the entry of various types of white blood cells (including lymphocytes, neutrophils, monocytes) into ischemic brain tissue, and these mechanisms together lead to ischemic stroke. Inflammatory response causes the release of cytokines and chemokines, which attracts more white blood cells and inflammatory cells to the lesion site, forming lipid plaques [86]. These plaques gradually increase and continue to deposit, and eventually form atherosclerotic plaques; this is the most important cause of ischemic stroke [87,88]. Therefore, the role of coagulation and inflammatory mechanisms in stroke treatment is complex and multifaceted, and further studies are needed to elucidate the specific mechanisms and therapeutic potential. Understanding these interactions is essential for developing effective therapeutic strategies that can target both coagulation and inflammation, potentially improving outcomes in a variety of clinical settings.

## 6. Therapeutic Implications

Ischemic stroke is a disease caused by thrombosis in the brain. Taking ischemic stroke as an example, intravenous thrombolysis with recombinant tissue-type plasminogen activator (rt-PA) administered within 4.5 h after stroke onset is currently the most effective stroke treatment [89,90]. However, the toxicity and reperfusion injury of rt-PA can increase the risk of brain damage and neuroinflammation [91,92]. Pharmacological research on stroke has always been a research hotspot, but there is not an efficient and safe full range of therapeutic drugs [93]. Table 1 shows the current clinical commonly used thrombotic treatment strategies.

### 6.1. Targeted Therapies for NETs

Research indicates that NETs play a significant role in both arterial thrombi (such as cerebral thrombi) and venous thrombi [95,96]. NETs can promote thrombus formation through multiple mechanisms, including enhancing platelet adhesion, activating coagulation factors, and inhibiting anticoagulant mechanisms. Current therapeutic strategies targeting NETs for thrombus treatment mainly include the following approaches, And in Table 2, the representative studies of thrombosis therapy targeting NET are listed:

**Targeting NET Degradation**

DNase-I therapy: DNase-I (deoxyribonuclease I) is an enzyme that degrades DNA. By breaking down the DNA scaffold of NETs, it can disrupt the structure of NETs, thereby reducing their thrombogenic potential. Studies have shown that DNase-I treatment can significantly inhibit thrombus formation [97]. In addition to DNase-I, other nucleases may also exert similar effects, although research in this area is currently limited.

**Targeting NET Stability**

Platelet factor 4 (PF4): PF4 can bind to NETs and enhance their resistance to DNase, thereby stabilizing NETs. This strategy can reduce the release of NET degradation products (such as cell-free DNA and histones), which are important factors in thrombus formation. By inhibiting the degradation of intact NETs, PF4 reduces the release of thrombogenic cell-free DNA, thereby lowering the risk of thrombus formation [98].

**Targeting the Mechanisms of NET Formation**

Inhibition of PAD4 enzyme: PAD4 (peptidylarginine deiminase 4) is a key enzyme in NET formation. Inhibiting PAD4 can reduce the release of NETs. Studies have shown that PAD4 inhibitors can significantly decrease NET formation, thereby reducing the risk of thrombus formation [99].

Inhibition of neutrophil activation: By inhibiting the activation of neutrophils, the release of NETs can be reduced. For example, inhibiting receptors on the surface of neutrophils (such as TLR4) or intracellular signaling pathways (such as NF-κB) can reduce NET formation [100,101].

**Targeting the Interaction Between NETs and Platelets**

Combination therapy with antiplatelet drugs: In some cases, NETs promote thrombus formation by adhering to platelets. Therefore, a combination of antiplatelet drugs (such as aspirin and P2Y12 inhibitors) with NET-targeting drugs may be more effective.

Targeting platelet surface receptors: By targeting receptors on the surface of platelets (such as glycoprotein GPIIb/IIIa), the adhesion of platelets to NETs can be reduced, thereby lowering the risk of thrombus formation [102].

**Targeting the Interaction Between NETs and the Complement System**

Complement inhibitors: The interaction between NETs and the complement system can promote thrombus formation [103]. Therefore, complement inhibitors (such as anti-C5 monoclonal antibodies) may reduce the prothrombotic effects of NETs by inhibiting the activation of the complement system.

**Other Strategies**

Gene therapy: Targeted knockout or modification of genes related to NET formation using gene-editing technologies (such as CRISPR-Cas9) may be a potential therapeutic strategy [104].

Immune regulation: By modulating the immune system to reduce excessive neutrophil activation, the release of NETs can be decreased [105].

**Table 2:** Thrombotherapeutic approaches targeting NETs.

Therapeutic Approach	Mechanism of Action	Application/Disease Context	Key References
DNase I Therapy	Degrades DNA backbone of NETs, destabilizing thrombi and reducing inflammation	Ischemic stroke, myocardial infarction	Shown to improve post-MI cardiac function and reduce NET-mediated damage [106,107].
PAD4 Inhibitors	Block histone citrullination by inhibiting peptidylarginine deiminase 4 (PAD4), suppressing NET formation	Acute MI, atherosclerosis	PAD4-deficient mice exhibit reduced NETs and attenuated cardiac injury [108].
Ion Channel Targeting (e.g., SK3, TRP)	Modulates calcium signaling and ROS production in neutrophils to inhibit NETosis	Myocardial infarction, ischemia-reperfusion injury	SK3 inhibition reduces NET formation and improves cardiac outcomes [109]
Anti-Histone Therapy	Neutralizes cytotoxic free histones released from NETs, protecting endothelial cells	Stroke, thrombotic microangiopathy	Anti-histone antibodies or CRP mitigate histone toxicity [110].
Colchicine	Inhibits microtubule polymerization, reducing neutrophil activation and NET release	Post-MI cardiac remodeling	Preclinical studies show reduced NETs and improved cardiac function [111].
ROS Pathway Targeting	Suppresses NADPH oxidase or scavenges ROS to block NETosis initiation	Arterial/venous thrombosis	ROS inhibition decreases NET release and thrombotic risk [112].
Gut Microbiota Modulation	Alters gut flora to reduce pro-inflammatory mediators, indirectly suppressing NETs	Myocardial ischemia-reperfusion injury	Specific gut microbiota exacerbate NET-driven injury; targeting microbiota improves outcomes [113].

### 6.2. Neutrophil Drug Delivery Systems

Inflammation is now recognized as one of the major targets for the development of novel stroke therapies with promising applications [114,115]. Jie Li et al. found that an elevated systemic inflammatory response index was associated with poor outcome in mild ischemic stroke [116], suggesting that reducing the post-thrombotic inflammatory response is necessary to reduce the disability rate of ischemic stroke and improve the prognosis of patients. However, the current treatment strategy for ischemic stroke lacks post-thrombolytic intervention for thrombus site inflammation, and patients are prone to be left with severe neurological damage that affects their future quality of life. Therefore, reducing the thrombus-induced inflammatory response during thrombolysis is a future concern in thrombosis therapy.

The blood–brain barrier (BBB) is an important protective mechanism for the brain, consisting of astrocytes, pericytes, cerebral microvascular endothelial cells, and the basement membrane [117,118], which effectively prevents harmful substances from entering brain tissue, but also poses a challenge for drug and therapeutic delivery. More than 98% of small-molecule drugs have difficulty entering the brain, resulting in a lack of effective clinical therapeutic strategies for treating central nervous system (CNS) injury and similarly hindering drug delivery to the CNS after ischemic stroke [119,120,121].

**Figure 6:**
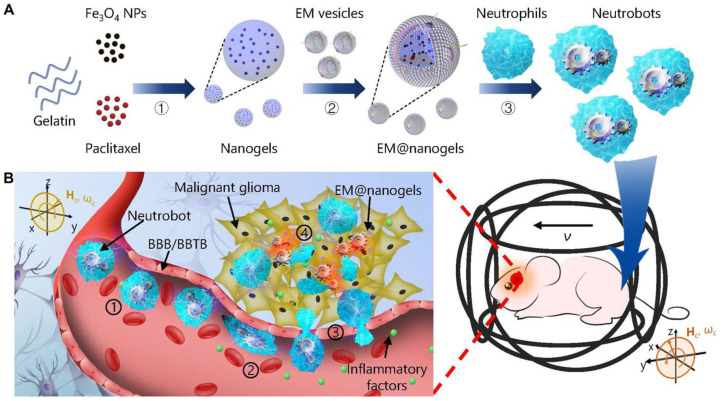
Schematic of active therapeutics for dual-responsive neutrobots in vivo. (**A**) Schematic of the three-step fabrication of the neutrobots. ① Synthesis of the Fe3O4 nanoparticles (Fe3O4 NPs) and Paclitaxel-loaded nanogels through emulsion/solvent evaporation method. ② Preparation of E. coli membrane-coated nanogels (EM@nanogels) by coextrusion technique. ③ Fabrication of neutrobots by phagocytosis of EM@nanogels into natural NEs. (**B**) Schematic of active delivery of dual-responsive neutrobots toward malignant glioma. ① Active accumulation of neutrobots close to glioma upon exposure to external magnetic field. BBB/BBTB, blood-brain barrier/blood-brain tumor barrier. ② Chemotaxis of the neutrobots along the gradient of inflammatory factors. ③ Penetration of the BBB of neutrobots through the natural capability of NEs. ④Paclitaxel release from the neutrobots toward malignant glioma. Hc, circular RMF; ωc, critical frequency of RMF; v, velocity of neutrobots. Reproduced with permission from Science Robotics, Copyright 2021, Zhang, H. [128].

Excessive aggregation of neutrophils can aggravate thrombus formation and cause further tissue damage, but neutrophils can penetrate the blood–brain barrier. In recent years, many studies have shown that neutrophils are an ideal drug delivery vehicle. Garanina AS et al. compared the efficacy of neutrophils as carriers of cancer nanotherapeutic agents, including liposomes, PLGA, and magnetic nanoparticles delivered to tumors. Neutrophil-mediated delivery has been shown to reduce tumor growth and improve animal survival [122]. Nanoparticle transport is achieved by targeting activated neutrophils by coating the nanoparticles with anti-CD11b antibodies and promoting neutrophil infiltration into the tumor by photosensitizer-mediated acute inflammation [123]. In recent studies, methotrexate (MTX), an immunosuppressive agent for the treatment of inflammatory and autoimmune diseases, was encapsulated in cationic liposomes (MTX-liposomes) and loaded into neutrophils by co-incubation to successfully deliver to inflamed skeletal muscle and ischemic heart tissue [124]. Shao et al. loaded neutrophils with bacterial membrane-camouflaged mesoporous silica nanoparticles (MSNs) by coculturing living neutrophils for inflammation-targeted drug delivery [125]. Zhang et al. constructed cross-linked dendrimer polylysine (DGL) nanoparticles containing catalytic enzymes and modified them with the neutrophil-targeting peptide Ac-PGP to deliver drugs to regions of cerebral ischemia through a neutrophil-mediated mechanism [126]. Xue et al. used neutrophils as cell carriers to load nanoparticles of cationic lipid polymers to target atherosclerotic sites [127]. Hongyue Zhang et al. developed a neutrophil-based robot that uses an external magnetic field to attract neutrophils to the brain. By taking advantage of the inflammatory chemotactic effect of neutrophils, targeted drug delivery to brain tissue was carried out to improve the local drug concentration in the affected area, and active targeted therapy for brain glioma by swimming micro/nanobots was realized. It has an inhibitory effect on brain glioma (Figure 6) [128]. Zhenyu Luo et al. reported that neutrophils can cross the bone marrow–blood barrier to increase drug concentrations in the bone marrow. In a bone metastasis cancer model, neutrophil delivery has been shown to significantly inhibit tumor growth [129]. Similarly, the ability of neutrophils to cross the blood–brain barrier could be used to deliver drugs, which could overcome the inability of small-molecule drugs to cross the blood–brain barrier.

Gene therapy strategies, such as viral vector-mediated gene delivery, genome editing technologies, and RNA-based strategies, are being investigated to reduce neuroinflammation [130]. The achievement of targeted delivery of genetic drugs requires overcoming three major challenges. First, the effective encapsulation of therapeutic agents is essential for the targeted delivery of gene drugs. For example, RNA drugs degrade rapidly when exposed to blood. Second, the biocompatibility and biodegradability of the delivery vector are essential for extending the lifespan of gene drugs in vivo. Third, penetration of various biological barriers, such as the blood–brain barrier, into areas of inflammation or disease is important for targeted delivery [131]. To address these issues, we propose the use of neutrophils as a carrier for targeted delivery of nanoparticle-encapsulated genetic drugs to the thrombus area. Neutrophils retain intact membrane structures and immunosuppressive antigens of native NEs, such as CD47. They can evade the attack of the immune system by interacting with various inhibitory receptors, such as signal regulatory protein α [132], thereby extending their lifespan in the circulatory system. The chemotactic movement of neutrophils along gradients of inflammatory factors inherits the chemotaxis of natural NEs, enabling neutrophils to move to sites of inflammation and cross the blood–brain barrier. Accurate delivery of drugs to ischemic brain tissue can reduce the inflammatory response and improve the recovery of patients with ischemic stroke.

**Figure 7:**
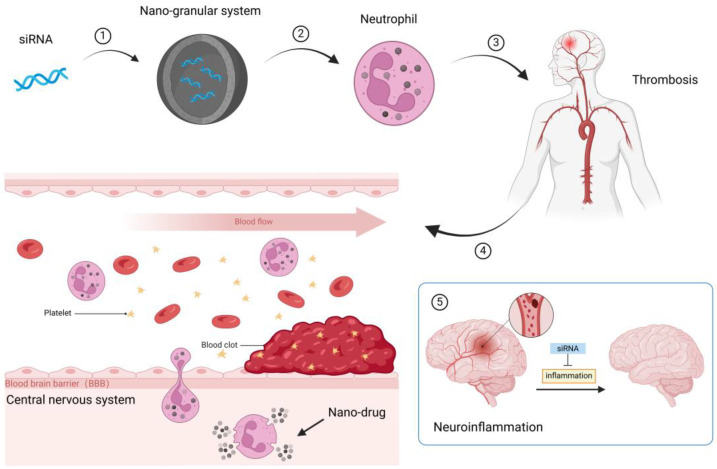
Neutrophil drug delivery system for the treatment of ischemic stroke. ① siRNA sequences targeting target genes (e.g., target validation of signaling pathways) are designed and then loaded into the nano-granular system. ② Nano-granular systems were loaded on patient-derived neutrophils. ③ Neutrophils containing nano-granular systems were infused back into patients with thrombosis. ④ Neutrophils move chemotactically on inflammation to reach the thrombus and cross the blood-brain barrier to release the drug. ⑤ The expression of the target gene was suppressed, the level of inflammation was reduced, and the nerve injury was alleviated. Figure created using Biorender.

Therefore, a genetic drug could be designed to attenuate the inflammatory response. After collecting neutrophils from patients, genetic drugs, such as siRNA targeting inflammatory pathways, can be loaded into neutrophils and then transfused back into the patient so that drug-loaded neutrophils can recruit to the thrombus area to release drugs and reduce the inflammatory response in the lesion area. This may prevent further recruitment of neutrophils, reduce brain tissue damage in stroke patients, accelerate recovery of stroke patients after injury, and improve patient prognosis (Figure 7).

## 7. Conclusions

Neutrophils emerge as central mediators bridging inflammation and thrombosis, orchestrating complex interactions that drive both physiological defense and pathological thrombogenesis. This review highlights the dual role of neutrophils in host immunity and disease progression, particularly through the release of NETs. NETs, composed of DNA, histones, and proteases, serve as a scaffold for platelet adhesion, coagulation factor activation, and thrombus stabilization, thereby amplifying thromboinflammatory cascades in conditions such as sepsis, stroke, and cardiovascular diseases. The interplay between neutrophils and platelets—mediated by receptor-ligand interactions (e.g., P-selectin/PSGL-1) and soluble mediators—creates a feedforward loop that exacerbates vascular occlusion and tissue damage. Mechanistically, NETs promote thrombosis by activating factor XII, degrading anticoagulants like TFPI, and enhancing endothelial dysfunction. These pathways are further modulated by inflammatory cytokines (e.g., IL-8, TNF-α) and biomechanical stressors, linking hemodynamic forces to neutrophil activation.

Current strategies for the treatment of thrombosis, including conventional thrombolytic therapy and anticoagulant therapy, face several limitations, such as the risk of bleeding and poor treatment of inflammatory lesions in thrombosis. Targeting neutrophil activity, such as by dissolving NETs or leveraging their chemotactic properties for drug delivery, offers promising therapeutic strategies for thrombotic and inflammatory diseases. Innovative approaches leveraging neutrophil biology—such as by dissolving NETs or leveraging their chemotactic properties to cross the blood–brain barrier for drug delivery—offer novel avenues for targeted intervention. These strategies capitalize on neutrophils’ inherent chemotactic properties to deliver therapeutics directly to thrombotic or inflammatory sites, minimizing systemic toxicity.

Future research should focus on using the natural chemotactic properties of neutrophils for drug delivery, exploring stable and efficient drug delivery methods, and exploring new therapeutic strategies to regulate the activity of neutrophils and NETosis in thrombotic diseases, thereby developing new clinical treatments for thrombosis and related diseases. Addressing these challenges will advance our ability to disrupt the neutrophil–inflammation–thrombosis axis, ultimately improving outcomes in thrombotic and inflammatory diseases.

In summary, this review underscores the need for a paradigm shift in thrombosis management—therapies that precisely modulate neutrophil activity and NET-driven pathways beyond conventional anticoagulation, offering hope for safer and more effective treatments in clinical practice.

## Figures and Tables

**Table 1:** Current clinical treatment strategies for thrombosis.

Methods of Treatment	Description	Advantages	Limitations	Commonly Used Drugs
Intravenous thrombolytic therapy	The active dissolution of already formed thrombus, by activating the fibrinolytic system in the body, degrades fibrin.	It can rapidly dissolve the thrombus and restore cerebral blood flow.	There are strict time window constraints. There is a risk of bleeding, especially intracranial hemorrhage, which is a serious complication.	Recombinant tissue plasminogen activator (rt-PA) and urokinase (UK), tenecteplase (TNK)
Endovascular mechanical thrombectomy	Direct physical removal of the thrombus from the occluded vessel can extend from 6 to 24 h after symptom onset, with a window that depends on the clinical and imaging characteristics of the patient.	The treatment time window should be extended to provide reperfusion therapy opportunities for more patients.	The procedure is complex, requires specialized skills, may damage the venous valves, and carries the risk of bleeding and vascular injury.	N/A
Fibrinolytic therapy	Reducing or disrupting the fibrin network reduces blood viscosity and the likelihood of thrombosis.	It can reduce platelet aggregation and prevent experimental thrombosis.	May induce immune antigenicity, need to pay attention to allergic symptoms.	Urokinase, streptokinase, etc.
Antiplatelet therapy	It blocks the initial steps of arterial thrombosis mainly by inhibiting the activation and aggregation of platelets.	It is used for the prevention and treatment of thrombosis in the arterial system, especially in atherosclerotic diseases, such as coronary heart disease, ischemic stroke, and peripheral arterial disease.	There is also a risk of bleeding, especially with long-term use.	Aspirin, clopidogrel, ticagrelor
Anticoagulant therapy	It mainly delays the blood coagulation process by inhibiting the key factors in the coagulation cascade (such as thrombin, factor Xa, etc.).	Anticoagulant therapy is mainly used for venous system diseases, which can effectively block the production of thrombin and reduce thrombosis.	Long-term use may increase the risk of bleeding [94].	Heparin, warfarin, new oral anticoagulants and so on.
Volume expansion therapy	For most patients with ischemic stroke, there are not enough randomized controlled trials (RCTS) to support the effect of volume expansion and hypertension on the prognosis.	It may increase cardiac output, improve oxygen delivery, and improve tissue perfusion.	It may cause overload hazards such as pulmonary edema, and volume expansion may not be beneficial in patients who are likely to have pulmonary edema or in whom volume expansion is ineffective.	N/A
Treatment of rehabilitation	It includes physical therapy and occupational therapy aimed at improving the patient’s motor function and increasing the patient’s independence in activities of daily living through exercise and training.	It is helpful to restore the ability of daily living and improve the quality of life of patients.	Long-term adherence is required, and the effect varies from person to person.	N/A
Preventing further stroke	These include lifestyle modifications and medications aimed at reducing the risk of further stroke.	Reducing the risk of further stroke by controlling risk factors.	Long-term adherence to medication and lifestyle changes is required.	N/A
